# Gearbox Fault Identification Model Using an Adaptive Noise Canceling Technique, Heterogeneous Feature Extraction, and Distance Ratio Principal Component Analysis

**DOI:** 10.3390/s22114091

**Published:** 2022-05-27

**Authors:** Cong Dai Nguyen, Cheol Hong Kim, Jong-Myon Kim

**Affiliations:** 1 Faculty of Radio-Electronic Engineering, Le Quy Don Technical University, Hanoi 10000, Vietnam; dainc@lqdtu.edu.vn; 2 School of Computer Science and Engineering, Soongsil University, Seoul 06978, Korea; cheolhong@ssu.ac.kr; 3 Department of Electrical, Electronics, and Computer Engineering, University of Ulsan, Ulsan 44610, Korea; 4 PD Technology Cooperation, Ulsan 44610, Korea

**Keywords:** fault diagnosis, feature extraction, gearbox fault identification, adaptive noise canceling technique, principal component analysis, support vector machine

## Abstract

Using an adaptive noise canceling technique (ANCT) and distance ratio principal component analysis (DRPCA), this paper proposes a new fault diagnostic model for multi-degree tooth-cut failures (MTCF) in a gearbox operating at inconsistent speeds. To account for background and disturbance noise in the vibration characteristics of gear failures, the proposed approach employs ANCT in the first stage to optimize vibration signals. The ANCT applies an adaptive denoising technique to each basic frequency segment in the whole frequency response of vibrations. Following that, a novel DRPCA is used to extract the discriminating low-dimensional features. The DRPCA initially determines each feature’s relative proximity to fault categories by computing the average Euclidian distance ratio between similar and dissimilar classes. The most discriminatory features with the lowest dimensions are selected, as determined by principal component analysis (PCA). The new DRPCA is created by combining distance ratio–based feature inspection with PCA. The optimal feature set containing the most discriminative features is then fed to the support vector machine classifier to identify multiple failure categories. The experimental results indicate that the proposed model outperforms the state-of-art approaches and offers the highest identification accuracy.

## 1. Introduction

Gear failures are the most common problems in gearboxes, which are used in many types of machinery, automobiles, and wind turbines [1,2,3,4], because of difficult and constant working conditions. A gearbox failure can cause severe system failures, financial losses, and workforce risks. Thus, the early detection of gearbox failings is essential. The condition-based monitoring method provides maintenance tasks based on gearbox data and can extend gearbox lifespans while reducing maintenance costs [5]. The most effective technique for diagnosing gearbox failure is vibration-based condition monitoring [6]. The gearbox vibration signal includes tooth meshing harmonics, sideband frequencies, and free oscillations. In the ideal case, failure-related oscillations are blended sideband frequencies [7,8]. However, system interconnections, mechanical systems (resonances caused by a shaft, bearing, gear, etc.), and background noise are major sources of interference [9,10]. Vibration signals have noisy components that dominate and distort the failure-related ingredients with their random magnitudes and occurrences. To identify gear failures, the raw vibration signal must be optimized to reduce uncertainty about protruding fault-associated elements, such as meshing frequency harmonics and sideband frequencies.

For rotating-machine fault diagnosis, many signal processing techniques have been proposed to examine the signal in multiple domains. The Hilbert transform (HT), window bandpass filters, short-time Fourier transform, empirical mode decomposition (EMD), and wavelet transform (WT) are some of those techniques [11,12,13,14,15]. Hybrid techniques use WT-HT and EMD-WT [16,17]. All those techniques reduce the interference noise to some extent, but they degrade the amplitudes of the sideband and meshing frequency harmonics, distorting the failure signature information associated with gear failures in the vibration signals. Therefore, those approaches are unlikely to be useful in identifying the various types of multi-degree tooth-cut failure (MTCF) in a gearbox using fluctuating speed operations. Nguyen et al. [18,19] demonstrated that their adaptive noise reduction method effectively reduced noise in a raw vibration signal. They used the resulting signal to identify failures in gearboxes with multi-level tooth cut faults. Their adaptive model works by searching an optimal set of Gaussian parameters linked to filter weights across the whole frequency range of a vibration signal. However, a non-stationary and complicated vibration signal can be caused by both random noise and gear stiffness variation. Meshing harmonics, sideband components, and random noise are all present in gearbox vibration signals, with different energy distributions in many frequency segments along the frequency spectrum. Therefore, applying a single optimized Gaussian parameter set to all frequency ranges is unsatisfactory. To analyze vibration signals from MTCF failures, we propose an adaptive noise canceling technique (ANCT). The proposed method adopts an adaptive denoising technique (ADT) from [18] to analyze the frequency domains segmentally. The optimized vibration signals provide a wealth of information about gear failures after they’ve been transformed by the ANCT. That is, the ANCT significantly reduces noise while retaining the original failure information. The proposed gearbox fault diagnostic approach uses the ANCT outputs to configure feature pools and identify failure types.

In terms of feature engineering and classification, the fault-relevant elements in the vibration signals of an MTCF gearbox are too similar because the failure categories are all reflected in the vibration characteristics in the same way. Thus, the output signals of the ANCT must be evaluated in an enhanced manner. Numerous approaches are used for feature extraction, including statistical feature calculation, complex envelope analysis, and wavelet packet analysis [20,21,22,23]. We hypothesize that expanding the discrimination representation of a failure diagnostic system beyond a single feature model will minimize the possibility of overlooking significant information in the data. In other words, we deem it preferable to generate as much information about the process state as possible, even if it is tainted by redundancy, and then filter out the most useful aspects in a subsequent information processing phase. Therefore, we use three feature computation techniques (statistics, wavelets, and envelopes) in this study to configure a heterogeneous feature pool (HFP). Although the HFP increases the effectiveness of gearbox fault expression for fault detection, the large dimensionality of the feature pool poses a difficulty for machine learning. To address that issue, feature selection [24], which is a critical technique in artificial intelligence, can be applied to reduce the dimensionality of the HFP and optimize system accuracy. This paper analyzes a collection of potential features using multivariate search methods, with the goal of minimizing duplication in the final, optimized feature set. Thus, we choose the most differentiating features from the HFP, which include both relevant and redundant information, by developing a search algorithm. From the many types of analysis available, including independent component analysis (ICA) [25], linear discriminant analysis (LDA) [26], genetic algorithm (GA) [27], and principal component analysis (PCA) [28,29], the PCA technique leverages eigenvalues from the covariance matrix of the variables to explore the statistical structures of the data and choose the principal elements (those with the highest eigenvalues). Both supervised and unsupervised PCA techniques were investigated in [29] for condition-monitoring of rotating machines. According to that analysis, PCA performed better than the other dimensional reduction techniques for failure condition classification. However, PCA does not consider the distance between the attributes of different failure classes. To address this issue, we propose a new feature selection approach, the distance ratio principal component analysis (DRPCA). This new technique starts by performing a PCA on the HFP to compute the average Euclidean distance parameters between features pertaining to the same and diverse failure categories. The novel DRPCA combines a relative distance–based evaluation with a PCA.

DRPCA configures a feature set of the most distinct features, which is then used as input data for machine learning–based classification. Compared with other artificial intelligence techniques, support vector machines (SVMs) have higher generalization, allowing them to diagnose mechanical defects in a rotating machine with high accuracy [30]. SVMs can also effectively separate nonlinear datasets using hyperplane mapping functions [31]. To classify multiclass data, SVMs use the one against one, one against all, or hierarchical strategies. The most accurate approach is a one against one SVM (OAOSVM) [32], which we thus use in our proposed model.

Moreover, gear fault diagnosis models have been proposed to identify the MTCF gearbox under variable speed conditions, and they validated the superiority of the classification accuracies [19,33]. These models were developed using a combination of adaptive denoising approaches and deep neural network architectures (DNAs). Accordingly, the results of the adaptive denoising process were then used to input to the deep neural network for the enhancement operation through three execution stages: (1) a high dimensional feature pool configuration by the automated feature extraction, (2) a fine-tuning process for selection of the most discriminative features, and (3) a gear fault identification process by the classifying layer in the deep network hierarchy. The constructed DNAs have effectively performed feature engineering and classification to obtain highly accurate classification outcomes. However, since DNAs use more computing resources than machine learning classifiers, they may not be appropriate for certain applications, particularly in real-time observation systems. Therefore, we propose the gearbox fault identification model to perform a manual feature engineering process (i.e., HFP + DRPCA) and a machine learning–based classification approach (OVOSVM) for achieving the precise identification of the fault categories.

The following outlines the contributions of this paper.

(1) ANCT is a new approach for signal processing that uses an adaptive method to identify the appropriate Gaussian parameter set for each basic frequency segment of a vibration frequency domain. The optimized vibration signal outputs of the ANCT contain failure-related information with decreased disturbance noise.

(2) Various feature models (statistical computation, wavelet basis decomposition, and complex envelope decomposition) are used to extract features from the ANCT results. The resulting HFP offers as many failure signatures as feasible for the condition monitoring process; it can also represent various fault types.

(3) The DRPCA chooses the most distinctive features in the HFP to identify fault categories. To compute the distance ratio between features of the failure types, the DRPCA first performs principal component analysis, examines the distance between the features of different failure types, and then selects the features with the highest relative distance ratio to create an optimal feature set with decreased dimensions.

(4) To evaluate the proposed technique, vibration signals from an MTCF gearbox with various types of failures were acquired from a real-world experimental testbed.

The remainder of this article is organized as follows: Section 2 discusses the underlying approaches used in this study; Section 3 explains the experimental testbed for an MTCF gearbox and vibration measurements; Section 4 constructs the proposed diagnostic model for an MTCF gearbox; Section 5 details the steps of performance assessment and the experimental results obtained from the proposed model; and Section 6 summarizes the findings of this study.

## 2. The Background of the Techniques

### 2.1. Adaptive Noise Filtering Technique

**Figure 1:**
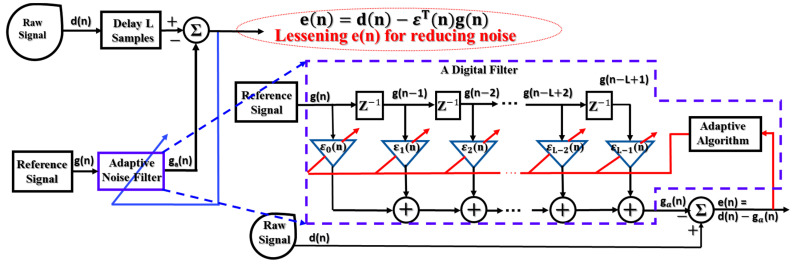
The functional structure of an adaptive noise filtering technique.

The adaptive noise filtering technique uses two key operational functions: digital filtering and adaptive processing [34], as shown in Figure 1. The adaptive method adjusts the coefficient parameters of a digital filter to reduce the output error for denoising.

Assuming that a coefficient vector of an L-tap finite impulse response digital filter is denoted by ε(n) (i.e., $\varepsilon \left(n\right)={\left[\varepsilon_{0},\varepsilon_{1},\ldots ,\varepsilon_{L-1}\right]}^{T}$), a raw signal d(n) and a reference signal g(n) (those are the discrete time signals, and n indicates a time variable in the discreteness) and output error e(n) can be calculated according to [34], as follows:

(1) $$e\left(n\right)=d\left(n\right)-\varepsilon^{T}\left(n\right)g\left(n\right)$$

The mean square error (MSE) is used to determine the convergence condition for the output error:

(2) $$J\;\equiv \;E\{e^{2}\left(n\right)\}=E\{{\left[d\left(n\right)-\varepsilon^{T}\left(n\right)g\left(n\right)\right]}^{2}\}\;\;J=\varepsilon^{T}\left(n\right)C\varepsilon \left(n\right)-2P\varepsilon^{T}\left(n\right)+E\left\{d^{2}\left(n\right)\right\}$$

where C≡E{g(n)d(n)} indicates the cross-correlation parameters of a reference signal and a raw signal, and $P\equiv E\left\{g\left(n\right)g^{T}\left(n\right)\right\}$ signifies the autocorrelation parameter of a reference signal. As shown in Equation (2), the MSE is a quadratic function, so there exists a unique global minimum corresponding to an optimal coefficient vector. The least mean square (LMS) process is the technique most often used to reduce the output error [35], and the weight parameters can be adjusted as follows:

(3) ε(n+1)=ε(n)+2λg(n)ε(n)

Here, the convergence factor (λ), which is used as a step-size parameter to estimate the minimum MSE, can be adjusted between about 0 and ${\left(LS_{r}\right)}^{-1}$ ($S_{r}$ is the average power of a reference signal g(n), and *L* signifies the order of a digital filter).

### 2.2. Wavelet Packet Transform (WPT)

WPT is a variant of the discrete wavelet transform that exhibits the capacity to decompose signal information in the high-frequency region in depth [36]. WPT divides a signal into several sub-bands, with great resolution in both the low- and high-frequency domains, by using a sequence of scaling analysis (low-pass) and wavelet analysis (high-pass) filters, resulting in $2^{l}$ sub-bands, where *l* is the depth level. The following formula encapsulates the WPT function [36]:

(4) $$W_{\delta ,\rho }^{n}\left(t\right)=2^{\frac{\delta }{2}}W^{n}\left(2^{\delta }t-\rho \right),\;$$

where ρ and δ are the transition and scale parameters, respectively. The scaling and mother wavelet functions are assigned as *n* = 0 ($W_{0,0}^{0}\left(t\right)=\Phi \left(t\right)$), and *n* = 1 ($W_{0,0}^{1}\left(t\right)=\Psi \left(t\right)$), respectively. Here, Φ(t) and Ψ(t) are a scaling function and a wavelet function that form the multi-resolution analysis technique in WPT. The scaling function represented by the low-pass filters is used to analyze the input signals in the low-resolution approximation spaces, while the wavelet function is applied to refine those approximation spaces in the higher frequency zones, implemented by the band-pass filters [36].

Then, other wavelet packet functions can be calculated through a recursive process, which is represented as

(5) $$W_{\delta ,\rho }^{2n}\left(t\right)=\sqrt{2}\sum_{\rho =-\infty }^{\rho =\infty }h\left(\rho \right)W^{n}\left(2t-\rho \right),\;$$

(6) $$W_{\delta ,\rho }^{2n+1}\left(t\right)=\sqrt{2}\sum_{\rho =-\infty }^{\rho =\infty }g\left(\rho \right)W^{n}\left(2t-\rho \right).$$

Here, h(ρ) and g(ρ) are low- and high-pass quadrate mirror filters, respectively, which are associated with the selected mother wavelet and scaling functions. Therefore, the wavelet coefficients of a signal s(t) obtained by the WPT method can be calculated by the following inner product formula:

(7) $$\theta_{\delta }^{n}\left(\rho \right)=<s\left(t\right),W_{\delta ,\rho }^{n}>=\int_{-\infty }^{\infty }s\left(t\right),W_{\delta ,\rho }^{n}\left(t\right)dt.$$

Using Equations (5)–(7), WPT decomposes each node into two sub-bands using high- and low-pass filters formed by the wavelet packet coefficients. Each coefficient $\theta_{\delta }^{n}\left(\rho \right)$ is the specific parameter for each resolution in the frequency domain and thus represents the scaling parameter and oscillation component in the observed signal.

### 2.3. Complex Envelope Analysis

The HT is used to analyze the complex envelopes of a signal to expand it into a complex space and generate an analytical signal in the new time domain. If *y*(*t*) represents the analytical signal of the observed signals *s*(*t*) in the time domain, then it can be expressed as follows [37]:

(8) $$y\left(t\right)=s\left(t\right)+j\tilde{s}\left(t\right),$$

where $j=\sqrt{-1}$, and $\tilde{s}\left(t\right)$ is defined as the convolution of *s*(*t*) and 1/πt in the time domain, as follows:

(9) $$\tilde{s}\left(t\right)=s\left(t\right)*\frac{1}{\pi t}=\frac{1}{\pi }\int_{-\infty }^{\infty }\frac{x\left(\tau \right)}{t-\tau }d\tau .$$

In the frequency domain, its frequency spectrum can be calculated as

(10) $$\tilde{S}\left(\omega \right)=-jsgn\left(\omega \right)S\left(\omega \right);-jsgn\left(\omega \right)=\{\begin{array}{c} j,\;with\;\omega >0\\ 0,\;with\;\omega =0\\ -j,\;with\;\omega <0 \end{array}\;$$

The complex envelope analysis is then performed by computing the attributes of the observed signal in the complex spaces defined by Equations (8)–(10).

### 2.4. Principle Component Analysis

PCA is a common procedure for shrinking the dimensionality of a feature pool by assessing the variances of many extracted features to identify which attributes have the largest effect on the data structure. By using this statistical technique, it is possible to exclude weaker-distinctive elements from a high-dimensional pool while retaining the most fault-related representational components (i.e., the dimensionality of the feature pool is reduced). In other words, the initial coordinate space of the observed data maps onto the direction of increasing variance. The computation process of PCA, which is presented in [38], is explained in detail by the following formulas.

Consider $S\in {\mathbb{R}}^{m\times p}$ as a feature vector with zero mean and unit variance that has been normalized from an original feature vector consisting of m rows of samples and p variable columns. The covariance matrix C can be computed as

(11) $$C=\frac{1}{m-1}S^{T}S$$

By performing single-value decomposition for matrix C, it can be represented as

(12) $$C=V\Delta V^{T},$$

where V is a matrix of eigenvectors of C, and Δ signifies a diagonal matrix, which consists of the downward order eigenvalues of C (i.e., $\lambda_{i}\geq \lambda_{i+1}\geq ..\geq \lambda_{p}\geq 0$). Then, by producing the transfer matrix $ℒ\in {\mathbb{R}}^{m\times k}$ (k<p) with k eigenvectors (k columns of V) equivalent to the desired number of primary eigenvalues (k), the dimension of the observed variables can be reduced (k<p). Therefore, several formulae represent the manipulating performance of a PCA:

(13) $$\lfloor \begin{array}{c} Κ=Sℒ\;\\ \;\hat{S}=Κ{ℒ}^{T}\;\\ E=S-\;\hat{S}\;\\ \;S=Κ{ℒ}^{T}+E. \end{array}$$

where Κ is a scoring matrix, ℒ is a loading matrix, $\hat{S}$ indicates a principal source of the original feature space, and E represents the redundancy in the process. It is essential to select k to filter the principal components.

## 3. The Experimental Gearbox Test-Rig and Dataset Description

**Figure 2:**
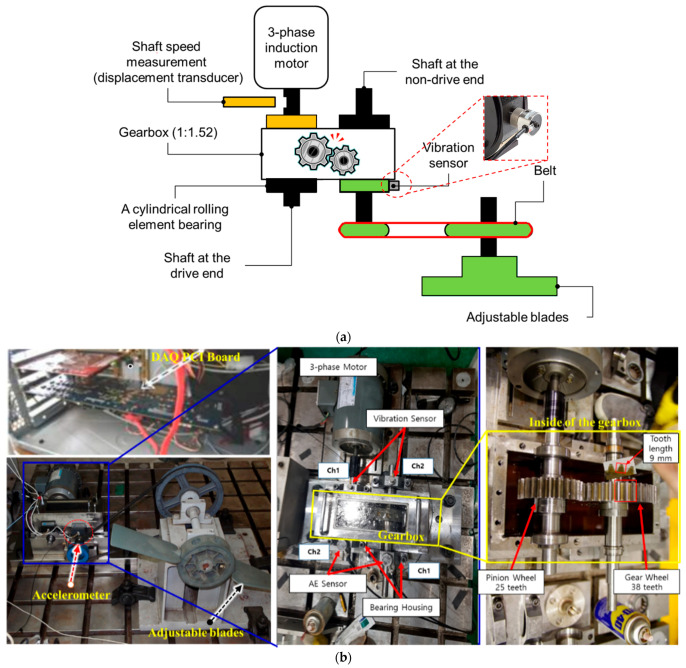
The experimental gearbox test rig: (**a**) functional structure block diagram and (**b**) actual arrangement of the test rig.

Figure 2 depicts the experimental test equipment used to obtain vibration data from a gearbox system. The functional structure of the test rig is shown in Figure 2a; the gearbox transfers rotational motion from a three-phase motor to adjustable blades (i.e., the load) through a 1:1.52 reduction ratio. The non-drive shaft is used to connect the load and a gear wheel. The driveshaft connects the pinion wheel directly to the three-phase motor. The pinion wheel and gear wheel are engaged by the teeth (each tooth is 9 mm in length), as indicated in Figure 2b. The vibration sensor is positioned at the non-drive end to monitor the vibration characteristics of the gearbox. The displacement transducer is mounted on the driveshaft to track the rotational speeds of the pinion wheel. The vibration dataset in this study was collected using a PCI-based data acquisition device that digitizes vibration signals from the accelerometer. Table 1 summarizes the specifications for the vibration sensor, transducer, and acquisition board.

**Figure 3:**
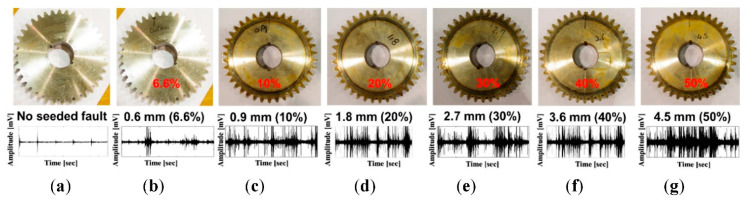
The multi-degree tooth cut failures of the gear wheel and the vibration signals at 600 RPM: (**a**) NF, (**b**) F1, (**c**) F2, (**d**) F3, (**e**) F4, (**f**) F5, and (**g**) F6.

Real-world gearbox systems suffer two major sorts of gear failures: those induced during manufacture or installation (e.g., incorrect tooth sketching, wheel alignment, or parallelism) and those that occur during operation (e.g., tooth pitting, tooth spalling, tooth cracking, tooth breakage). Gear failures were simulated in this testbed by cutting one tooth with several degrees of tooth length (9 mm) to generate multi-degree tooth cut failures of 6.6% (0.6 mm), 10% (0.9 mm), 20% (1.8 mm), 30% (2.7 mm), 40% (3.6 mm), and 50% (4.5 mm), as shown in Figure 3. These failures were generated to approximate the operational problems that occur in actual gearbox systems as a result of long-term rotational performance fatigue.

Analog vibration signals were produced by an accelerometer and transformed into digital vibration samples at a sampling frequency of 65.536 kHz. Each sample was one-second long, and we captured 65,536 data points. The acquisition method was performed 150 times for each of the seven failure conditions and alternated over four rotational speeds (300 RPM, 600 RPM, 900 RPM, and 1200 RPM), which produced the design for the experimental dataset used in this study (Table 2).

**Figure 4:**
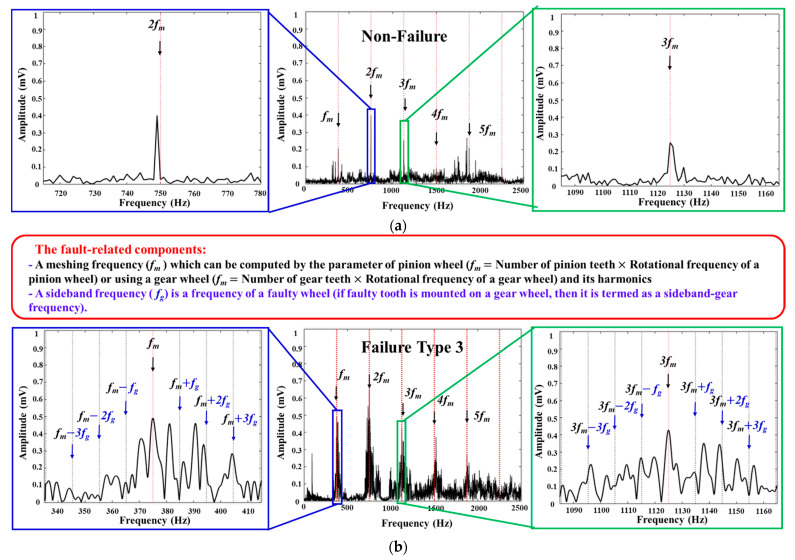
The frequency spectrum analysis of vibration samples acquired from the non-failure (**a**) and failure type 3 (**b**) conditions at 900 RPM.

The vibration samples gathered fully reflect the vibrational characteristics of the gearbox. Specifically, a linear and regularly oscillating vibration signal is derived from a gearbox system in the absence of failure [39]. As helpful identifying information, the frequency spectrum comprises the tooth meshing frequency (which reflects the stiffness of the gearbox) and its harmonics. When a pair of gears rotates across a defective tooth, the angular velocity changes abruptly. When a gearbox fails, its rigidity fluctuates, complicating the signal structure (e.g., making it nonlinear and nonstationary). In other words, the nonlinear and nonstationary properties of a faulty gearbox are shaped by its angular acceleration, as recorded by a vibration sensor. The frequency spectrum of the vibration signals contains the meshing frequency harmonics, sideband frequency tones surrounding each harmonic, and other oscillations, which can each exhibit amplitude-phase modulation characteristics [40]. The frequency spectrum analysis of vibration samples taken from a gearbox rotating at 900 RPM under non-failure and failure type 3 conditions is shown in Figure 4.

## 4. The Proposed Gearbox Fault Diagnosis Model

**Figure 5:**
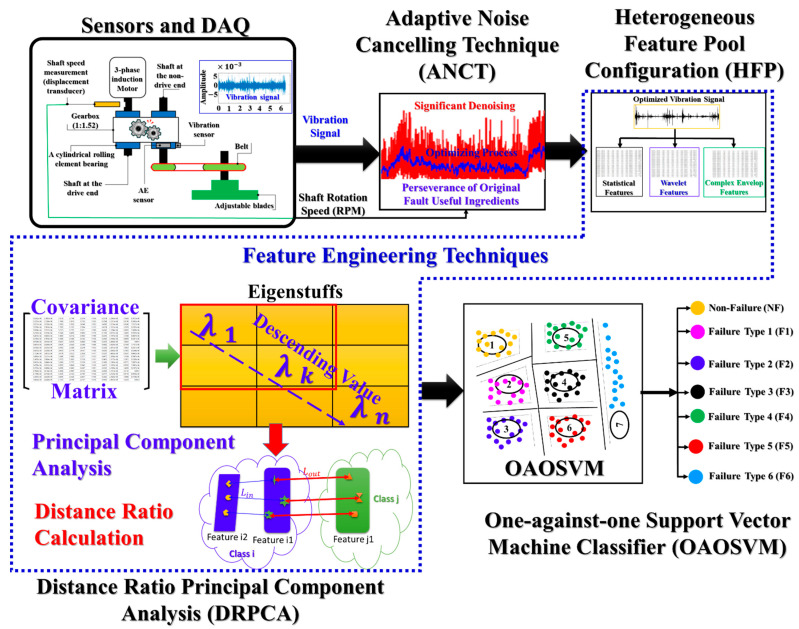
The processing flow and functional blocks in the proposed gearbox fault diagnosis method.

The novel fault diagnosis approach presented in this article was used to categorize seven different types of gear failure in an MTCF gearbox running at various rotational speeds. The processing flow shown in Figure 5 is defined by the function blocks applied. The data acquisition system described in Section 3 collected vibration signals to create the dataset for the experiment. Each vibration sample was collected by sampling at the high frequency of 65.536 kHz for one second (the time required to complete a few rotation cycles) to generate a rich sample and capture as much failure-related information as feasible. The ANCT module processes those raw vibration signals. In the first step, a low-pass filter and three-times down-sampling of the raw vibration signals [18] are used to produce vibration samples with real operating frequency tones from 0–10 kHz, which the accelerometer can detect (Table 1). This step attempts to eliminate redundancy in the frequency range of the raw vibration signal introduced by the high-speed sampling procedure. Later, as shown in Figure 5, effective denoising approaches, multiple domain feature extraction, DRPCA-based feature selection, and an SVM classifier are used to construct the proposed failure identification model. The following sub-sections contain complete functional descriptions of those modules.

### 4.1. ANCT

**Figure 6:**
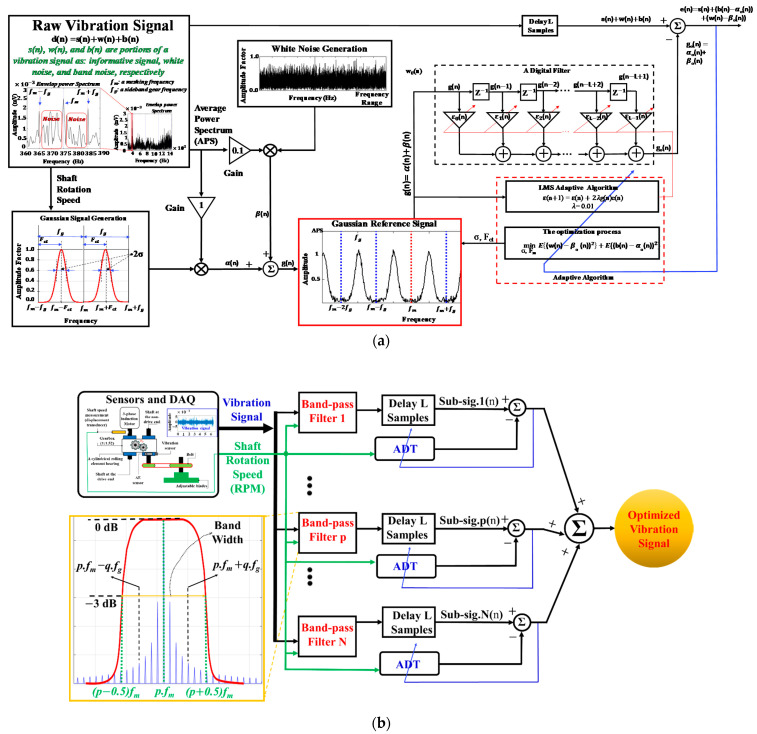
The processing flows and functional blocks of the ADT method (**a**) and the ANCT (**b**).

The ANCT is based on the adaptive noise filtering technique outlined in Section 2 and is used to reduce the noise and redundancy of a vibration signal. Accordingly, the raw signal input is the vibration signal, and the reference signal is composed of the signal that simulates the noise oscillations in a gearbox vibration signal, termed the noise-pretended signal (NPS) (Figure 1). Two essential steps operate in the ANCT: the generation of NPSs and the execution of adaptive algorithms to access and reduce noise in multiple segments of the vibration frequency domain. The adaptive denoising techniques (ADTs) developed and verified in [18,19,33] have shown outstanding results in removing noise and protecting the failure-related signal. The noise in the gearbox vibration signal is categorized into two forms using those methods: white noise caused by the measuring system (e.g., data acquisition systems, electronic devices) and waveband noise induced by unrelated mechanical component resonances. Thus, as shown in Figure 6a, the NPS is formed by combining two functions of a uniform random distribution to emulate white noise and a Gaussian distribution to imitate the analogous behavior of waveband noise. Additionally, the parameters (i.e., mean and standard deviation) of the Gaussian signal, which are functions of the rotating speed variable, are associated with filter weights to be adjusted by the LMS adaptive algorithm [19]. The following formula describes the Gaussian function part of the NPS:

(14) $$G_{\mathrm{gs}}\left(m\right)=\sum_{m=1}^{M}e^{-\frac{{\left(m-F_{ct}\right)}^{2}}{2\sigma^{2\;}}}.$$

where M denotes the number of sideband frequency segments or meshing frequency harmonics. $F_{ct}$ and σ are, respectively, the mean and standard deviation, which are proportional to the rotational speed of the shaft. As a consequence, when a gearbox rotates at different speeds, a Gaussian signal is created that can provide access to the frequency gap between two successive sideband frequency tones.

Next, the LMS technique is used to adaptively adjust the parameters ($F_{ct}$, σ) of each generated NPS in conjunction with the filter weights to achieve the optimized parameter set [18]. Figure 6a illustrates the signal processing flow and function blocks of the ADT method. As shown in Figure 6a, the overall procedure of the ADT for each vibration sample is to determine the general optimal parameter set for an NPS coupled to an optimal filter coefficient vector for its full frequency domain (0–10 kHz). Nonetheless, the frequency spectrum of a vibration signal is composed of several basic frequency segments (BFSs), which have different energy distributions. Thus, we propose the ANCT to segment the vibration frequency domain into numerous BFSs (the segment width of each BFS equals a meshing frequency value and is centered on a meshing frequency harmonic) and then apply the ADT method [19] to each BFS to obtain an optimal parameter set for each BFS, thereby producing a series of optimized parameter sets for each vibration signal. The ANCT output is the summation of all the optimized sub-signals, which have been adaptively denoised using the series of segment-observed optimal parameter sets. Thus, the frequency range of the resultant signal of the ANCT is reconstructed in the same range as the input vibration signal. In that way, the ANCT can outperform previous ADT approaches in terms of noise reduction effectiveness and maintaining the original failure-related frequency tones for heterogeneous feature extraction in subsequent processes. The function block schematic of the ANCT is shown in Figure 6b. The ANCT partitions a vibration signal into N sub-signals, each with a frequency spectrum of one BFS (i.e., the frequency range of each sub-signal, which equals the range of BFS or the value of a meshing frequency), using N band-pass filters (Filter 1, Filter 2, …, Filter p, …, Filter N) simultaneously. Each filter is an IIR Chebyshev Type-I bandpass filter [41] with an order of 30, a bandwidth that is equal to the meshing frequency, and a band-pass frequency range in the consecutive series of the frequency domain of a vibration signal. N is the quotient of the maximum frequency of vibration signal and the meshing frequency. The ADT is then applied to each sub-signal to produce optimally segmented sub-signals. The output signal of the ANCT is then formed by averaging the N-optimized sub-signals. This output signal is referred to as the *optimized vibration signal* and is used to configure the HFP to explore failure-related information across a multiplicity of observation domains.

### 4.2. Heterogeneous Feature Pool Configuration

#### 4.2.1. Statistical Feature Calculation

The optimized vibration signal, i.e., the output of the ANCT, mostly contains intrinsic defective symptoms of the MTCF gearbox and is statistically computed and extracted in the two domains of time and frequency [42]. The calculation produces twenty-one features—three features in the frequency domain and eighteen features in the time domain—for each optimized vibration signal. Those features might contain some discriminant failure features that can be deemed subservient or congruent in the feature selection stage, as described in Table 3.

#### 4.2.2. Wavelet Package Decomposition (WPD)

**Figure 7:**
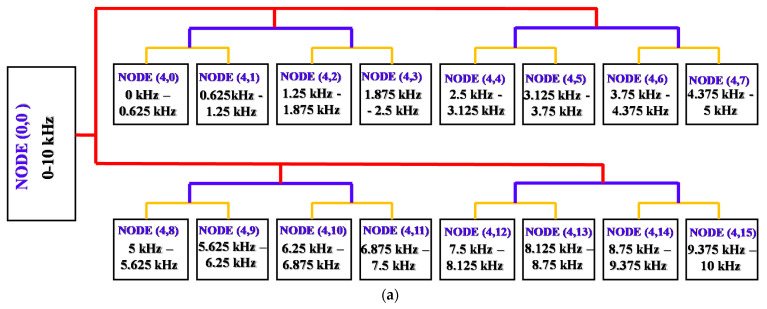

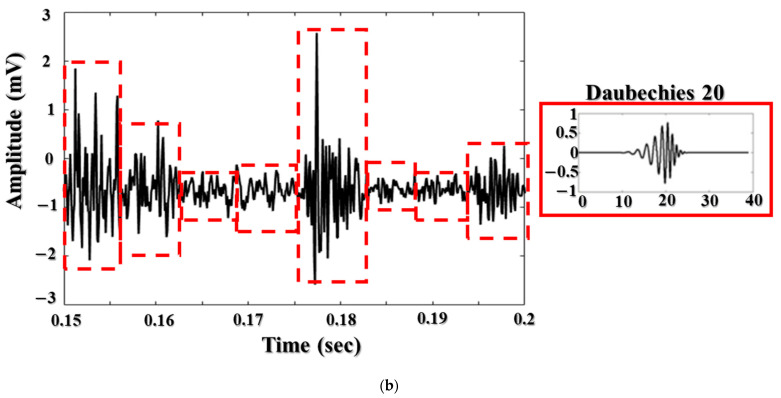
Four-level wavelet package decomposition (**a**) and the analogous oscillation patterns of a vibration signal and the Daubechies 20 function (**b**).

As shown in Section 2, WPT is more efficient than other wavelet techniques in analyzing signals in both the low- and high-frequency regions. The entropy and relative energy of the nodes, decomposed by WPT, reveal significant information about the impulsive oscillations in a vibration signal [36] and indicate the reflected vibration of various gear failure conditions. In general, the most difficult aspects of WPD are finding and choosing the mother function as well as the level of the vibration signal decomposition. The feature extraction results are greatly influenced by the selection. The method to find the best wavelet function for analysis of a vibration signal in a rotating machine fault diagnosis system, which was proposed by Rafiee et al. [43], demonstrated that the Daubechies family functions are more effective than other family functions, and the optimal level value is four. Therefore, in this study, we use WPD to execute four-level WPT and produce sixteen wavelet nodes (Figure 7a), which contribute wavelet-based features to the HFP by computing the entropy and relative energy of each node using the following two equations [44].

(15) $$RE_{node}\left(k\right)=\frac{\sum_{l=1}^{L}\alpha_{kl}^{2}}{\sum_{p=1}^{P}\sum_{l=1}^{L}\alpha_{pl}^{2}}$$

(16) $$E_{node}\left(k\right)=-\sum_{l=1}^{L}p_{k}\left(l\right).log_{2}\left(p_{k}\left(l\right)\right);where\;p_{k}\left(l\right)=\frac{\alpha_{kl}^{2}}{\sum_{l=1}^{L}\alpha_{kl}^{2}}\;\;$$

where $RE_{node}\left(k\right)$ and $E_{node}\left(k\right)$ are the relative energy and entropy of node *k*, respectively. L and P signify the total number of wavelet coefficients (i.e., $\alpha_{kl}$) for each node and the number of all decomposed nodes (P = 16), respectively.

The proper mother function of Daubechies 20 exhibits oscillation behaviors. These approximately homologous oscillations can find the resonance of the tiny variations in vibration, which are characteristic of the investigated failure types, as illustrated in Figure 7b. As a result, the WPD uses Daubechies 20 as the mother wavelet function to decompose each optimized vibration signal into 32 wavelet-based features for the HFP.

### 4.3. The Novel Distance Ratio Principal Component Analysis

Thus, multiple domain extraction from the output signal of the ANCT is used to configure the HFP in a high-dimensional feature space. The cardinality of the HFP is 123:21 statistical characteristics, 32 wavelet features, and 70 complex envelope features. However, the HFP contains redundant attributes that cannot be used to distinguish the different types of MTCF gearbox defects and therefore reduce the classification accuracy of the diagnostic model. To solve this problem, we propose a new DRPCA approach for choosing the discriminative features that express the fault type specifications. The DRPCA is processed in the following stages.

*Stage 1*. The average Euclidian distance ($L_{in}$) to features of the same failure type is calculated [45]:

(17) $$L_{in}=\frac{1}{P.Q}\sum_{p=1}^{P}\sum_{q=1}^{Q}D_{Euc}\left(p,q\right);$$

*Stage 2*. According to [45], the average Euclidian distance ($L_{out}$) between a specific feature vector of failure type *i* and other feature vectors of failure type *j* (i≠j, and 1≤i,j≤T) is computed:

(18) $$L_{out}=\frac{1}{P.T.Q}\sum_{t=1}^{T}\sum_{p=1}^{P}\sum_{q=1}^{Q}D_{Euc}\left(t,p,q\right);$$

*Stage 3*. The distance ratio by normalizing the quotient of the two distances attained from Equations (17) and (18) is determined:

(19) $$R_{d}=\frac{\frac{L_{out}}{L_{in}}}{\max\left(\frac{L_{out}}{L_{in}}\right)}.$$

where $D_{Euc}\left(u,v\right)=\sqrt{\sum_{k=1}^{K}{\left(u_{k}-v_{k}\right)}^{2}}\;$ is the Euclidian distance between two feature vectors; P is the total number of features (P=123, the dimension of the HFP); T denotes the number of failure types (T = 7); and Q signifies the observed sample count for each failure type. From Equation (19), $R_{d}$ is stated in the range of [0, 1] and enlarges as $L_{out}$ increases and $L_{in}$ decreases. This tendency indicates the specification of discriminative features because it results in less variation within a single failure type and much higher variation across different types.

*Stage 4*. Apply a PCA (explained in Section 2.4) to search the features with $R_{d}\geq 0.9$:

(20) $$\mathrm{DRPCA}=PCA\left(optimal\;features\left(R_{d}\geq 0.9\right)\right).$$

The DRPCA provides a collection of the most advantageous features, which are the most discriminative features in the HFP, to configure a new feature pool, dubbed the *optimal feature set*.

### 4.4. Multi-Class Support Vector Machine to Identify Failure Conditions

SVMs were first developed to classify binary data on the basis of statistical processes and quadratic function learning theory. They work by looking for the special space with the biggest separation between two binary classes in the observed dataset [46].

We assume the need to train for a binary dataset with N samples of {($\alpha_{n}$, $\gamma_{n}$), n = 1, 2, …, N}, where $\alpha_{n}\in {\mathbb{R}}^{Q}$, Q is the dimension of a feature vector, and the category labels are $\gamma_{n}$ ($\gamma_{n}\in \left\{-1,+1\right\}$). The special space, designated by w, can be obtained by increasing the margin width and decreasing the structural risk, as in the following expression [47]:

(21) $$\left(w,b\right)=\underset{w,b}{\mathrm{argmin}}\frac{1}{2}w^{T}w+R\sum_{n=1}^{N}\zeta_{n},\;$$

subject to $\gamma_{n}(w^{T}\Phi \left(x_{n}\right)+b)\geq \;1-{\;\zeta }_{n}$ and ∀n = 1, 2, …, N; $-{\;\zeta }_{n}\leq \;0$, ∀n = 1, 2, …, N, where b denotes bias; $\zeta \;=\left\{\zeta_{1},\zeta_{2},\ldots ,{\;\zeta }_{N}\right\}$ indicates a collection of slack variables; R is the trade-off coefficient; and $\Phi \left(x_{n}\right)$ is the expanded representation space of the feature vectors. By applying the Lagrange duality method [48] using Lagrange multipliers ($\mu_{n}$, $\mu_{k}$), the solution for Equation (21) can be transformed into the following:

(22) $$\underset{\mu }{\mathrm{Argmax}}w\left(\mu \right)=\sum_{n=1}^{N}\mu_{n}-\frac{1}{2}\sum_{n=1}^{N}\sum_{k=1}^{N}\mu_{n}\mu_{k}\gamma_{n}\gamma_{k}\Phi^{T}\left(\alpha_{n}\right)\Phi \left(\alpha_{k}\right)$$

subject to: $\sum_{n=1}^{N}\mu_{n}\gamma_{n}=0$, $0\;\leq b_{n}\leq \;R$, ∀n = 1, 2, …, N. Here, $\alpha_{n}$ and $\alpha_{k}$ are feature vectors of the input training data, which can be mapped into a new feature space with a larger dimensionality using the function $ℱ\left(\alpha_{n},\alpha_{k}\right)=\Phi^{T}\left(\alpha_{n}\right)\Phi \left(\alpha_{k}\right)$, which is called a kernel function, such as a sigmoid, Gaussian, radial base, linear, or polynomial function. To categorize the dataset into several classes (this article uses seven failure classes), the initial binary SVM can be improved by using one of several network architectures, including hierarchical, one-against-all, and one-against-one. The OAOSVM architecture showed reliable classification capability [32,49]. Thus, we applied it to identify the MTCF failure types.

## 5. Performance Evaluation Results and Discussion

In this section, we evaluate the performance of the proposed diagnosis model for an MTCF gearbox under varying speed conditions through three key processes: signal processing, feature pool configuration, and discriminant feature–based failure type identification.

### 5.1. The Effectiveness of the ANCT Performance

**Figure 8:**
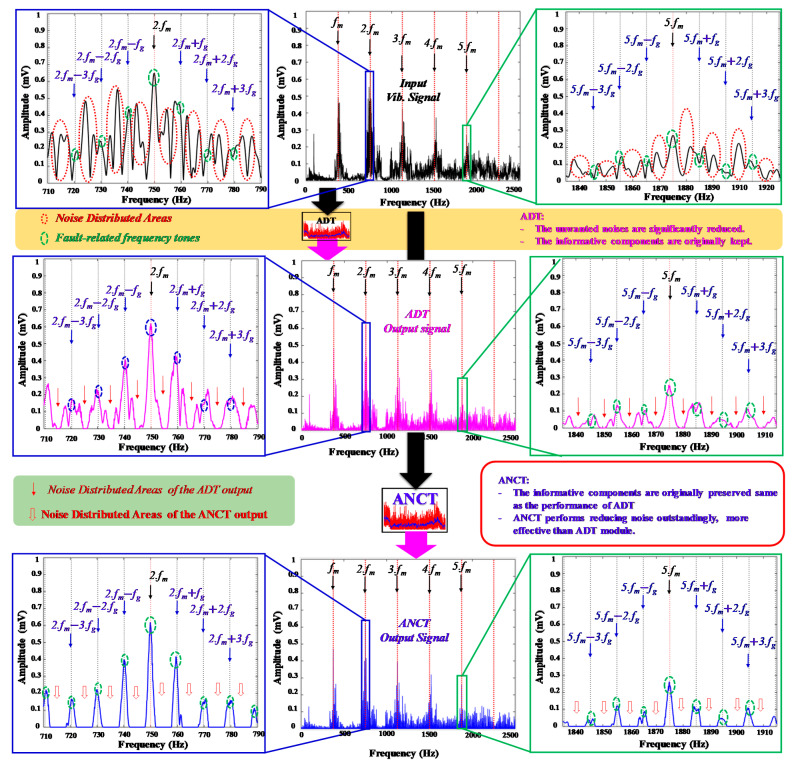
Visualized comparison of the ADT and ANCT performance.

Compared with conventional methods such as the HT, WT, window bandpass filter, and empirical mode decomposition, the ADT approach proposed in [18] demonstrates superior ability to process gearbox vibration signals to reduce noise and preserve useful fault information. Thus, the proposed ANCT was developed to enhance the denoising performance of the ADT by partitioning a vibration spectrum into many BFSs and then optimizing the vibration signal for each BFS using the segmented optimum NPS and filter coefficient set obtained from the ADT technique. To validate the efficacy of the ANCT method, the spectrum analysis of three vibration signals is shown in Figure 8: a raw vibration signal, an ADT output, and an ANCT output. In Figure 8, the dotted green circles represent items associated with failure-related frequency tones that were previously redeemed in the ADT and ANCT outputs. Furthermore, the ANCT has a considerably greater denoising capacity than the ADT; the noise regions in the ANCT output (shown by the dotted red circles) are far more degraded than those in the ADT output. As a result, ANCT outperforms ADT and other conventional signal processing techniques. In summary, the ANCT achieves considerable noise reduction while preserving the failure-related signatures, in this case the tooth meshing frequency harmonics and sideband frequency tones, in the raw signals. ANCT processed the vibration data of an MTCF gearbox with seven different failure categories over four different rotation speeds to provide optimized vibration signals. Those signals were then used to configure the HFP (which produces 123 features for each sample), as described in Section 4.2.

### 5.2. DRPCA-Based Feature Selection and Classification Results

**Figure 9:**
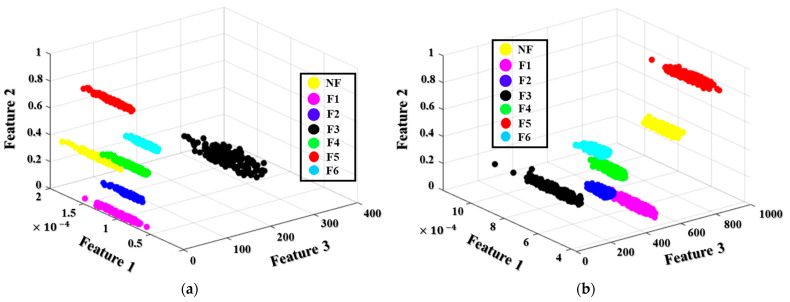

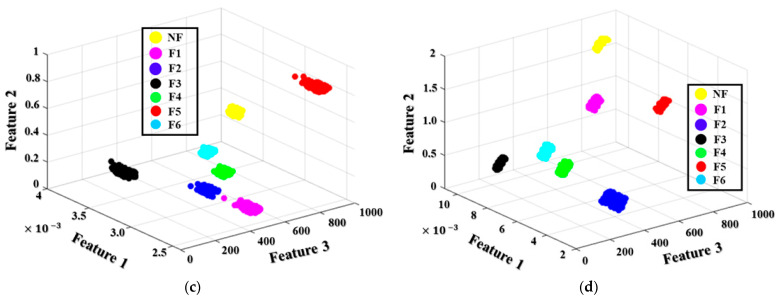
Three-dimensional visualization of samples of the seven failure types under four rotational speeds: (**a**) 300 RPM, (**b**) 600 RPM, (**c**) 900 RPM, and (**d**) 1200 RPM.

The applied diagnostic model uses a novel DRPCA procedure to capture the most discriminant features from the HFP in a smaller dimension. The PCA technique, as discussed in Section 2.4, is used to identify the importance of dynamically connected parameters or principal components, represented by a covariance matrix of eigenvectors. The PCA approach was used in this experiment to identify the attributes that produce primary elements with an eigenvalue greater than 80%. This value was chosen to capture the informative features that associated with the first 30 principal components, whose eigenvalues were greater than the remainder of the components. Experimental assessments in [28,50] reveal that the features with the greater eigenvalues contain the most fault-related information in the sequence of the all input features. The resulting principal components are then estimated using the distance ratios (according to the process given in Section 4.3). As a result of computing the DRPCA, features with substantial distance ratios ($R_{d}\geq 0.9$) are selected as the most discriminative features. The new optimal feature set is composed of three outcome features (designated Feature 1, Feature 2, and Feature 3), which resulted from a statistic model, wavelet decomposition, and complex envelope analysis, respectively. In Figure 9, a 3D figure based on those three optimal features illustrates the spatial distribution zones of samples of the seven MTCF failure states under the four different rotation speeds. As shown in Figure 9, the samples belonging to the same category are clustered together, and the samples belonging to different fault types are clearly separated in the feature space. The distance ratio thresholds are experimentally selected to obtain the number of the resultant features that are capable of representing failure types separately. The visualized spatial distribution of the categories in Figure 9 demonstrates that the threshold ($R_{d}=0.9$) is an appropriate selection for configuring the optimal feature subset of the most discriminative features in this study. The DRPCA output is then used as the input data for the OAOSVM classifier, which categorizes the data according to the failure types (i.e., NF, F1, F2, F3, F4, F5, and F6).

The classification evaluation of the proposed model was implemented using two experiments. In the first experiment, the optimal feature sets of all the samples of the seven failure types under four rotation speed conditions were combined to create a new feature set with the size of 4200 × 3. That new feature set was then randomly split into a training set and a testing set using a ratio of 7:3, respectively, to investigate the general capability of the proposed model. The second experiment was run to assess the robustness of the proposed gearbox fault diagnosis method in identifying failure types under inconstant rotational speed conditions. Therefore, the speed-related datasets (i.e., optimal feature sets) were used differently for the training set (two speed-related datasets) and the testing set (a single speed-related dataset). For instance, a training set was created by merging two speed-related datasets (300 RPM and 600 RPM for a dimension of 2100 × 3), and then a testing set was configured using the 900 RPM dataset (for a dimensionality of 1050 × 3). The four speed-related datasets (300 RPM, 600 RPM, 900 RPM, and 1200 RPM) were alternately selected to configure the training and testing sets for the classifier, creating four classifying executions.

K-fold cross validation (KCV) was used to estimate the generalized performance [51] of the proposed classifier. The training set was randomly dispensed into K subsets (K = 10 in this study), and then the OAOSVM classified K times based on K subsets to estimate the average classification accuracy (some of the K subsets were used for the training process, and the rest were used for the validation process). The average classification accuracy of the training model with KCV was calculated using the outcomes of the proposed training model on a validation set:

(23) $$C_{\mathrm{KCV}}=\frac{1}{K}\sum_{k=1}^{K}\left(\sum_{t=1}^{T}\frac{S_{t,k}^{\mathrm{TP}}}{S_{k}}\right)\times 100\%$$

where $S_{k}$ is the whole quantity of samples in a validation set at the kth iteration; $S_{t,k}^{\mathrm{TP}}$ is the number of true positives (i.e., samples of failure type *t* that are correctly assigned to category *t*).

**Figure 10:**
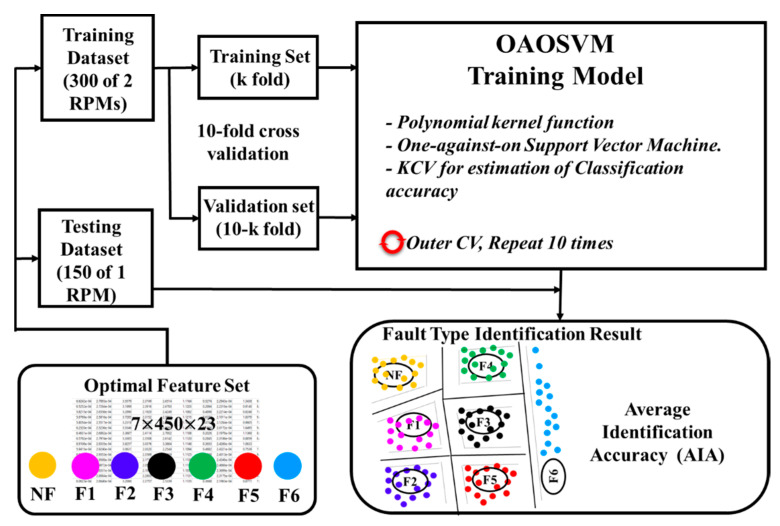
The learning model architecture using the one-against-one SVM.

The proposed fault diagnosis model was trained for various times (N) with the training set and testing sets of two experiments, and then the average identification accuracy (*AIA*) was summarized using Equation (24):

(24) $$AIA=\frac{1}{N}\sum_{n=1}^{N}\frac{\sum_{t=1}^{T}S_{t,n}^{\mathrm{TP}}}{S_{n}}\times 100\%,$$

where the whole quantity of true positives for failure type *t* at the nth evaluation process ($S_{t,n}^{\mathrm{TP}}$) and the total number of the testing set for the nth process ($S_{n}$) (the output results from the testing process) are used to calculate *AIA.* The proposed learning model architecture using an OAOSVM is depicted in Figure 10.

To validate the efficacy of our novel fault diagnosis method, we compared its performance with those of conventional fault diagnosis models, including M1 (ADT + HFE + DRPCA + OAOSVM), M2 (ANCT + HFE + PCA + OAOSVM), M3 (ANCT + HFE + GA + OAOSVM), M4 (ANCT + HFE + ICA + OAOSVM), M5 (ANCT + HFE + LDA + OAOSVM), M6 (LHTIS + HFE + DRPCA + OAOSVM), and M7 (WSET + HFE + DRPCA + OAOSVM). M1 is constructed by replacing the ACNT module of the proposed method with the ADT [18], which outperformed conventional signal processing methods for gearbox vibrations signals to explore the effect of noise reduction on the classification process. The other models were used to examine the ability of the DRPCA-based feature selection method by changing the proposed framework to use simple PCA (M2), GA [45] (M3), ICA (M4), and LDA (M5) instead of DRPCA. In addition, the other vibration signal analysis methods were applied as a replacement for the ANCT module in the proposed framework to establish models of M6 and M7. The local maximum high order time iterative synchro-squeezing (LHTIS) [52] and wavelet-based synchro-extracting transform (WSET) [53] techniques were used in M6 and M7, respectively.

The classification results of the proposed and reference models are tabulated in Table 4. The *AIA* results of M1 showing 14.31–24.37% are less than those of the proposed method. This is because the random disturbance noise elements in the vibration signals disorganize the feature engineering process and cause misclassification. ANCT provides better denoising performance than that specified in Section 5.1, demonstrating its high efficiency. In addition, when other signal processing approaches (LHTIS and WSET) are replaced with ANCT, the classification accuracies of the models are significantly lower than those of the proposed identification framework, showing 46.49–51.2% of M6 and 30.88–53.5% of M7. Furthermore, our DRPCA-based feature selection method outperforms M2 (13.75–31.9%), M3 (8.1–14.5%), M4 (17.79–30.18%), and M5 (17.83–36.57%), which verifies that the discriminant fault representation features of the optimal feature pool are very important for distinguishing the failure types of an MTCF gearbox under variable operating speeds. Overall, DRPCA is a suitable feature selection method for establishing the gearbox fault diagnosis model in this paper.

### 5.3. Discussion

The proposed model outperforms conventional state-of-the-art methods for identifying the failure types of an MTCF gearbox operating in inconstant speed conditions, yielding an average identification accuracy of 100% through two experiments. Denoising complex gearbox vibration data using a robust technique is critical for effective condition monitoring systems. Disturbance noise has a detrimental effect on feature engineering and identification performance. We developed our novel fault diagnosis model for an MTCF gearbox operating at changing speeds using ANCT for denoising, HFP and DRPCA for configuring the optimal feature set, and an OAOSVM classifier. During research and testing, our proposed approach achieved the highest classification performance, demonstrating its applicability.

In order to precisely identify the MTCF failure types in a gearbox system, the disturbance noise in the non-linear and non-stationary vibration signals should be eliminated in the first stage. As illustrated in Figure 4, the noise frequency tones are distributed in the whole range of the frequency spectrum, especially in the vicinity of the defect frequency tones. The high noise amplitudes might lead to misclassification of the failure categories and a deterioration in fault identification accuracy. The key technique of the ADT and ANCT methods is to use the adjustable Gaussian windows to adaptively access a zone between two consecutive sideband frequency tones along whole vibration spectrum to remove noise elements. Experimental results show that the ADT and ANCT outperforms the other conventional signal analysis approaches in [18] as well as M6 and M7 in reducing noise and preserving the failure-related ingredients. In addition, the proposed ANCT outperforms ADT in term of noise elimination, as shown in Figure 8. The next step of the fault diagnosis development process is the feature engineering and classification approach. In this paper, manual feature extraction and feature selection techniques are utilized to identify the most discriminant features that can be used as input to the machine learning–based classification algorithm. Experimental results show that the combination (HEF + DRPCA + OAOSVM) is the optimal solution for identifying seven MTCF gearbox failure types under different speed conditions, as shown in Table 4.

**Figure 11:**
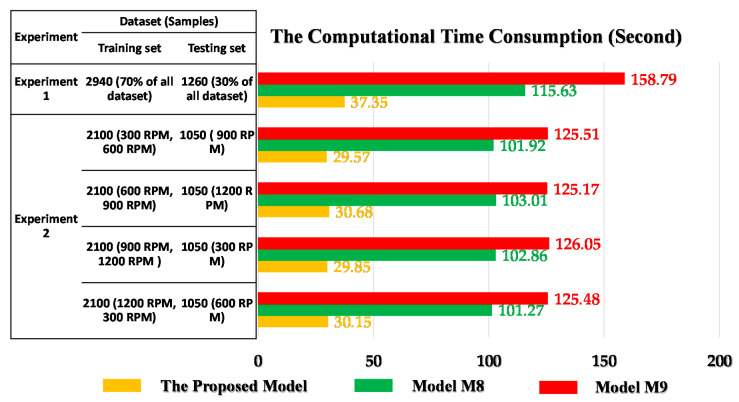
Visual expression of the computational time in the comparison of three models: the proposed model, M8, and M9.

On the other hand, the deep neural network architectures are also applied for automated feature extraction, feature selection, and classification in the posterior phase of the signal analysis. The sensitive and stable fault diagnosis frameworks are proposed by combining ADT and deep learning architecture; the stacked spare autoencoder-based deep neural network (SSA-DNN) in [19] (this model is referred to as model M8) and the deep convolutional neural network (DCNA) in [33] (M9) provide successful results (100%) for the identification seven failure types of an MTCF gearbox under variable speed operations. However, the computational time of the deep learning architectures in M8 and M9 requires more time than that of the proposed model (ANCT + HFE + DRPCA + OAOSVM), as shown in Figure 11.

## 6. Conclusions

In this paper, a novel model was proposed for identifying the fault types of multi-degree tooth-cut failures (MTCFs) in a gearbox system operating under inconsistent speed conditions.

(1) Vibration signals acquired from gearboxes have noisy components that dominate and distort the failure-related signal information, such as meshing frequency harmonics and sideband frequencies. To extract this information, a novel adaptive noise reduction method, ANCT, was proposed. ANCT uses an adaptive method to identify the appropriate Gaussian parameters for each segment of the vibration frequency domain. ANCT significantly reduces the noise in the vibration signal while retaining maximal failure-related information.
(2) Since multi-level tooth-cut failures are essentially the same type of faults that only differ in their relative size, a heterogeneous feature pool was constructed by calculating more than a hundred statistical parameters of the denoised vibration signal in multiple domains using wavelet packet decomposition and complex envelope decomposition to ensure the collection of maximal information on each type of MTCF fault.
(3) To reduce the dimensionality of the feature pool and select the most discriminative features for identifying the MTCF faults, a novel feature selection method, DRPCA, was proposed. DRPCA combines principal component analysis with relative distance ratio analysis of features of different fault types. The optimal feature set is constructed by selecting features with the highest relative distance ratio. This provides lower dimensionality, thereby improving the diagnostic performance of the OAOSVM, which was employed as a classifier.
(4) Finally, the performance of the proposed methodology was evaluated using a real-world experimental testbed and two different experiments based on the operational speed, where the vibration data were collected. In the first experiment, datasets for all the operational speeds were merged into a single set. Then, training and test subsets were constructed by randomly collecting features from this set. In the second experiment, the training dataset consisted of data recorded with one speed, while the testing dataset consisted of data recorded with other speeds. The proposed model outperformed the state-of-the-art approaches, with an average identification accuracy of 100% in both experiments. Moreover, our proposed model showed three times the speed-up over the relevant models, including the deep neural architectures.

## Figures and Tables

**Table 1:** The specifications of the sensors and the acquisition module.

Devices	Specification
Vibration sensor (Accelerometer 622B01)	Sensitivity (V/g): 10.2 mV/(m/s^2^)
	Operational frequency range: 0.42 to 10 kHz
	Resonant frequency: 30 kHz
	Measurement range: ±490 m/s^2^
4-Channel data acquisition PCI-based board	18-bit 40 MHz AD conversion, sampling frequency of 65.536 kHz is used for each of two channels simultaneously
Displacement transducer	Distance from the head of the transducer to a hole: 1.0 mm
	Hole diameter: 12.80 mm
	Sensitivity: 0 to −3 dB
	Frequency response: 0–10 kHz

**Table 2:** The vibration dataset for the MTCF gearbox obtained from the test rig and data acquisition equipment.

Gearbox Failure Condition	Description	Number of Samples for Each Rotation Speed (RPM)				Sampling Frequency (Hz)
		300	600	900	1200	
Non-Failure (NF)	Normal or perfect gearbox	150	150	150	150	65,536
Failure Type 1 (F1)	6.6% of tooth length (0.6 mm/9 mm)	150	150	150	150	65,536
Failure Type 2 (F2)	10% of tooth length (0.9 mm/9 mm)	150	150	150	150	65,536
Failure Type 3 (F3)	20% of tooth length (1.8 mm/9 mm)	150	150	150	150	65,536
Failure Type 4 (F4)	30% of tooth length (2.7 mm/9 mm)	150	150	150	150	65,536
Failure Type 5 (F5)	40% of tooth length (3.6 mm/9 mm)	150	150	150	150	65,536
Failure Type 6 (F6)	50% of tooth length (4.5 mm/9 mm)	150	150	150	150	65,536

**Table 3:** The statistical features extracted in the time and frequency domains.

Features	Equations	Features	Equations	Features	Equations
Peak	Max(|s|)	Shape factor	$\frac{s_{rms}}{\frac{1}{N}\sum_{n=1}^{N}\left|s_{n}\right|}$	Mean $\left(\bar{s}\right)$	$\frac{1}{N}\sum_{n=1}^{N}s_{n}$
Root mean square	$\sqrt{\frac{1}{N}\sum_{n=1}^{N}s_{n}^{2}}$	Entropy	$-\sum_{n=1}^{N}p_{n}.log_{2}\left(p_{n}\right)$	Shape factor square mean root	$\frac{s_{srm}}{\frac{1}{N}\sum_{n=1}^{N}\left|s_{n}\right|}$
Kurtosis	$\frac{1}{N}\sum_{n=1}^{N}\left(\frac{s_{n}-\bar{s}}{\sigma }\right)$	Skewness	$\frac{1}{N}\sum_{n=1}^{N}{\left(\frac{s_{n}-\bar{s}}{\sigma }\right)}^{3}$	Margin factor	$\frac{\max\left(s\right)}{s_{smr}}$
Crest factor	$\frac{\mathrm{Max}\left(\left|s\right|\right)}{s_{rms}}$	Square mean root	${\left(\frac{1}{N}\sum_{n=1}^{N}\sqrt{\left|s_{n}\right|}\right)}^{2}$	Peak to peak	max(s)-min(s)
Clearance factor	$\frac{\mathrm{Max}\left(\left|s\right|\right)}{s_{smr}}$	5th normalized moment	$\frac{1}{N}\sum_{n=1}^{N}{\left(\frac{s_{n}-\bar{s}}{\sigma }\right)}^{5}$	Kurtosis factor	$\frac{Kurtoris}{s_{rms}^{4}}$
Impulse factor	$\frac{\mathrm{Max}\left(\left|s\right|\right)}{\frac{1}{N}\sum_{n=1}^{N}\left|s_{n}\right|}$	6th normalized moment	$\frac{1}{N}\sum_{n=1}^{N}{\left(\frac{s_{n}-\bar{s}}{\sigma }\right)}^{6}$	Energy of signal	$\sum_{n=1}^{N}s_{n}^{2}$
Frequency center (FC)	$\frac{1}{N_{f}}\sum_{f}^{N_{f}}S\left(f\right)$	Root mean square frequency	$\sqrt{\frac{1}{N_{f}}\sum_{f}^{N_{f}}S{\left(f\right)}^{2}}$	Root variance frequency	$\sqrt{\frac{1}{N_{f}}\sum_{f}^{N_{f}}{\left(S\left(f\right)-FC\right)}^{2}}$

Here is an input signal (i.e., optimized sub-band), *N* is the total number of samples, *S*(*f*) is the magnitude response of the fast Fourier transform of the input signal s, $N_{f}$ is the total number of frequency bins, $\sigma =\sqrt{\frac{1}{N}\sum_{n=1}^{N}{\left(s_{n}-\bar{s}\right)}^{2}}$, and $\;p_{n}=\frac{s_{n}^{2}}{\sum_{n=1}^{N}s_{n}^{2}}$

**Table 4:** Diagnostic performance results from the proposed and reference models in different experiments.

Experiment	Dataset (Samples)		Average Identification Accuracy (AIA%)							
	Training Set	Testing Set	Proposed	M1	M2	M3	M4	M5	M6	M7
Experiment 1	2940 (70% of dataset)	1260 (30% of dataset)	100	75.63	72.79	85.50	81.68	70.18	62.70	68.54
Experiment 2	2100 (300 RPM, 600 RPM)	1050 (900 RPM)	100	80.31	72.54	86.65	69.82	63.43	63.51	46.50
	2100 (600 RPM, 900 RPM)	1050 (1200 RPM)	100	79.17	81.87	89.51	72.85	75.31	61.39	51.29
	2100 (900 RPM, 1200 RPM)	1050 (300 RPM)	100	81.71	86.25	87.30	81.21	67.62	48.80	69.12
	2100 (1200 RPM, 300 RPM)	1050 (600 RPM)	100	85.69	68.10	91.90	75.49	82.17	52.10	55.37

## Data Availability

The data are industrial. Due to the privacy policy of the industry involved, the data are not available publicly.

## Nomenclature

ANCT	adaptive noise-canceling technique
MTCF	multi-degree tooth-cut failures
HFP	heterogeneous feature pool
EMD	empirical mode decomposition
ADT	adaptive denoising technique
LDA	linear discriminant analysis
SVM	support vector machines
WPT	wavelet packet transform
KCV	K-fold cross validation
DRPCA	distance ratio principal component analysis
PCA	principle component analysis
HT	Hilbert transform
WT	wavelet transform
ICA	independent component analysis
GA	genetic algorithm
OAOSVM	one-against-one SVM
DAQ	data acquisition
AIA	average identification accuracy
